# Identification of risk factors for myocardial injury in acute ischemic stroke with diabetes mellitus: a retrospective cohort study on stroke-heart syndrome

**DOI:** 10.3389/fstro.2025.1617937

**Published:** 2025-07-23

**Authors:** Huijuan Pu, Yumin Wang, Guoping Zhao, Binbing Shi, Ni An, Changxi Zhang, Jie Liu, Wanling Wu, Hong Zhu, Lei Li, Defeng Pan

**Affiliations:** ^1^Department of General Practice, The Affiliated Hospital of Xuzhou Medical University, Xuzhou, China; ^2^Department of Cardiology, The Affiliated Hospital of Xuzhou Medical University, Xuzhou, China; ^3^Department of Geriatric Medicine, The Affiliated Hospital of Xuzhou Medical University, Xuzhou, China

**Keywords:** stroke-heart syndrome, acute ischemic stroke, diabetes mellitus, myocardial injury, risk factors

## Abstract

**Background:**

Ischemic stroke (IS) causes significant death and disability. Stroke-Heart Syndrome (SHS) involves cardiovascular complications, worsening outcomes. Diabetes mellitus (DM) increases the risk of myocardial injury following IS. This study explores risk factors for myocardial injury in acute ischemic stroke (AIS) with DM patients to improve early identification and prevention.

**Methods:**

This is a retrospective cohort study. Inclusion criteria: neuroimaging-confirmed AIS, admission within 72 h, and measured cardiac troponinT (cTnT) levels. Exclusion criteria included acute hemorrhagic stroke, other cTnT elevation causes, severe organ failure, infections, malignancies, and missing data. Logistic and LASSO regression analyses identified independent risk factors for myocardial injury.

**Results:**

Myocardial injury occurred in 194 patients. Independent risk factors identified were coronary heart disease (CHD), insular cortex lesions, peak brain natriuretic peptide precursor (peak NT-proBNP), C-reactive protein (CRP), and higher National Institutes of Health Stroke Scale (NIHSS) scores. These factors were significantly associated with myocardial injury and ROC analysis showed that the AUC for CHD was 0.621, the AUC for insular cortex lesions was 0.648, the AUC for NIHSS score was 0.726, the AUC for peak NT-proBNP was 0.816 and the AUC for CRP was 0.764. Subgroup analysis suggested that reperfusion therapy was associated with increased myocardial injury risk in various patient subgroups.

**Conclusion:**

CHD, insular cortex lesions, peak NT-proBNP and CRP levels, and higher stroke severity (NIHSS score) are significant risk factors for myocardial injury in AIS patients with DM.

## 1 Introduction

Stroke, is a disease caused by acute disorder of cerebral blood circulation and the third leading cause of death worldwide (Tadi and Lui, 2025; GBD 2021 Stroke Risk Factor Collaborators, 2024). It is estimated that by 2030, ~22 million people will die or suffer from disability due to stroke (GBD 2021 Stroke Risk Factor Collaborators, 2024; Pu et al., 2023). Among the different types of stroke, of which ischemic stroke (IS) is the most prevalent of type, accounting for ~65.3% stroke (GBD 2021 Stroke Risk Factor Collaborators, 2024).

Recent studies have highlighted a close relationship between the central nervous system and the cardiovascular system. Stroke not only directly impacts brain function but also exerts profound effects on the heart through neuroendocrine pathways, a phenomenon known as Stroke-Heart Syndrome (SHS) (Sposato et al., 2020). The SHS can be classified into 5 main categories: (1) ischemic and non-ischemic acute myocardial injury presenting with elevated cardiac troponin (cTn), which is usually asymptomatic; (2) post-stroke acute myocardial infarction (AMI); (3) left ventricular (LV) dysfunction, heart failure (HF), and post-stroke Takotsubo syndrome (TTS); (4) electrocardiographic changes and cardiac arrhythmias including post-stroke atrial fibrillation (AF); and (5) post-stroke neurogenic sudden cardiac death (Sposato et al., 2020). The pathophysiology of SHS is primarily related to autonomic nervous dysfunction, neurohormonal disturbances, and immune-inflammatory responses, which can increase cardiac load and subsequently accelerate myocardial cell injury (Ishiguchi et al., 2024). Studies have shown that SHS is associated with poor functional outcomes and is the second leading cause of death during the first few weeks following a stroke (Scheitz et al., 2018). Given the strong correlation between acute ischemic stroke (AIS) and cardiac events, the American Heart Association/American Stroke Association guidelines recommend the measurement of cardiac troponin (cTn) as a biomarker of myocardial injury in all patients with suspected stroke (Powers et al., 2018). The incidence of myocardial injury in patients with ischemic stroke can be as high as 27.2% (Mihalovic et al., 2023). Previous studies have shown that myocardial injury is associated with poor outcomes and increased mortality (Hess et al., 2025). Therefore, preventing and treating post-stroke myocardial injury is crucial for improving the prognosis of stroke patients.

Identifying and addressing the risk factors for myocardial injury are crucial for the prevention and treatment of post-stroke myocardial injury. Previous studies have indicated that the occurrence of myocardial injury in stroke patients is influenced by various factors, including age, a history of cardiac complications, metabolic disorders, and infarct location, which may be key determinants of myocardial injury (Johansen et al., 2024). Diabetes mellitus (DM), as a common chronic metabolic disease, plays a decisive role in triggering cardiovascular and cerebrovascular events (Maida et al., 2022). A meta-analysis has shown that the risk of IS in DM patients is 2.27 times higher compared to non-diabetic individuals. Moreover, DM patients have a 2- to 4-fold increased risk of developing cardiac events following a stroke compared to non-diabetic patients (Chen et al., 2016). Previous studies have indicated that diabetes is a major risk factor for acute myocardial infarction and IS, both of which are closely associated with atherosclerosis. However, in the Honolulu Heart Program's prospective study, the relative risk of ischemic thromboembolic stroke in DM patients was found to be 2.0 (95% CI, 1.4–3.0), even when other atherosclerosis related conditions, such as hypertension, hypercholesterolemia, and sedentary lifestyle, were strictly controlled, the impact of DM on stroke remained significant. Consequently, DM has been identified as an independent risk factor for IS (Maida et al., 2022).

The mechanism by which diabetes increases stroke is not fully understood. One possible mechanism is that DM patients often experience autonomic neuropathy, which may lead to an imbalance in the cardiac autonomic nervous system, thereby increasing the risk of myocardial injury (Lin et al., 2020). Additionally, insulin resistance and hyperinsulinemia, common in DM patients, may result in metabolic disturbances in the heart, further impairing cardiac function (Chen et al., 2017). Furthermore, DM patients are generally in a heightened inflammatory state with elevated oxidative stress levels, which may exacerbate myocardial damage (Yan et al., 2020).

Considering the close relationship between DM and cardiovascular events, this study aims to investigate the potential risk factors for myocardial injury in patients with AIS and concomitant DM. The findings could provide scientific evidence for the early identification and prevention of risk factors associated with post-stroke myocardial injury.

## 2 Methods

### 2.1 Study population

**Figure 1:**
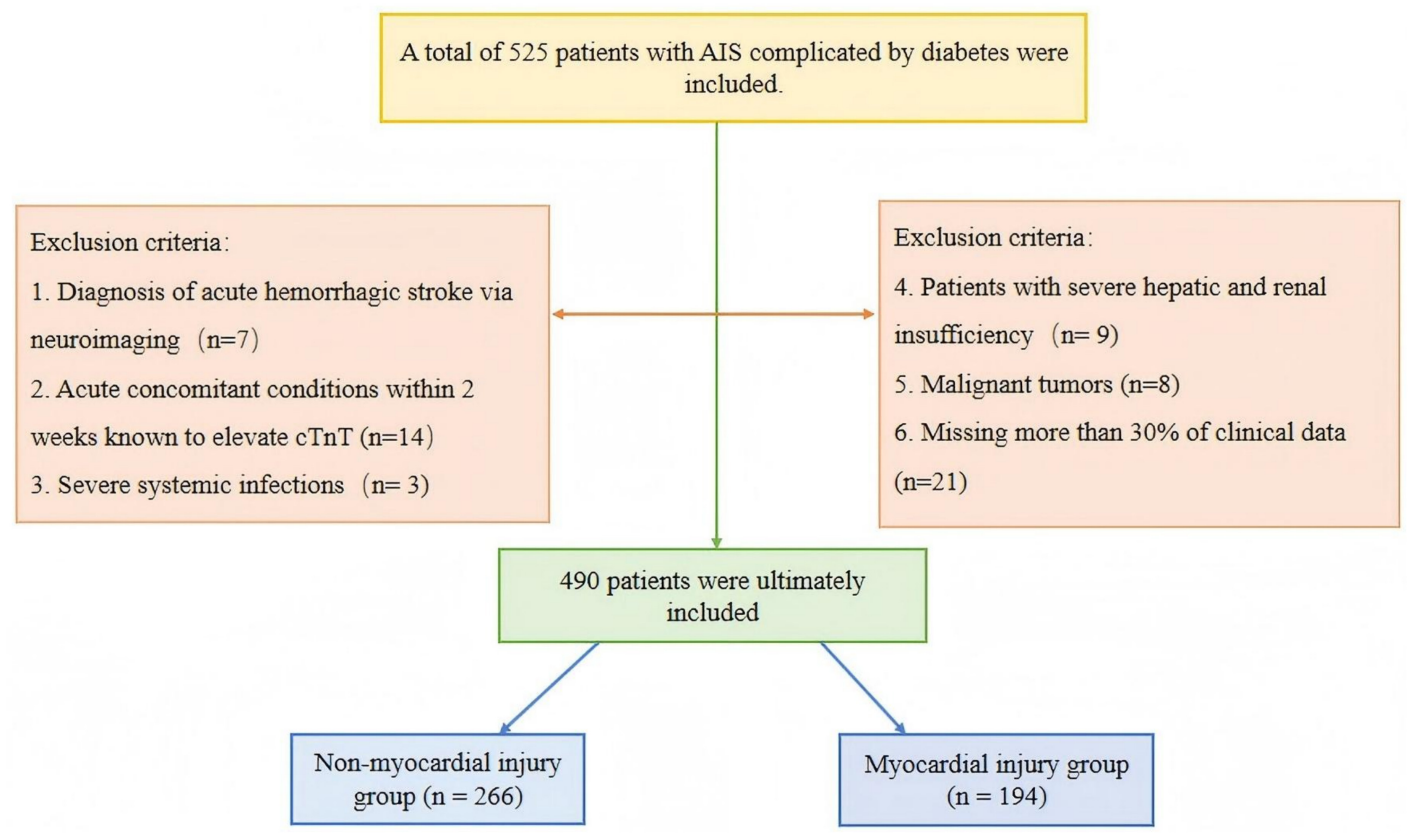
Study enrolment flowchart.

We retrospectively identified patients with AIS who were admitted to the Affiliated Hospital of Xuzhou Medical University between January 2021 and December 2023. AIS diagnosis was made according to the 2019 guidelines (Powers et al., 2019). The inclusion criteria were as follows: (1) Diagnosis of AIS based on neuroimaging; (2) Hospital admission within 72 hours of symptom onset; (3) Measurement of cardiac troponin T (cTnT) upon admission; (4) Patients with elevated cTnT upon admission, with at least one additional cTnT measurement within 48 h. The exclusion criteria were: (1) Diagnosis of acute hemorrhagic stroke via neuroimaging (Greenberg et al., 2022); (2) Acute concomitant conditions within 2 weeks known to elevate cTnT, including acute myocardial infarction, worsening of congestive heart failure, major cardiac surgery, sepsis, acute kidney injury, rhabdomyolysis, pulmonary embolism, and infective endocarditis; (3) Severe systemic infections; (4) Severe hepatic or renal insufficiency, defined as liver dysfunction (Child-Pugh class C or MELD score ≥ 15) or kidney dysfunction (end-stage renal disease on dialysis or eGFR < 15 mL/min/1.73 m^2^); (5) Malignant tumors; (6) Missing more than 30% of clinical data (Figure 1).

### 2.2 Data collection

Patient demographic data [age, sex, and body mass index (BMI)], traditional cardiovascular risk factors (hypertension, smoking, and alcohol consumption), comorbidities [coronary heart disease (CHD) and prior stroke], previous medication use (focusing on antiplatelet agents, anticoagulants, β-blockers, and statins), stroke treatment modalities (conventional drug therapy, thrombolysis, and mechanical thrombectomy), neurological function as assessed by the National Institutes of Health Stroke Scale (NIHSS) (Adams et al., 1999) to evaluate stroke severity, and the location of the ischemic lesion in the insular cortex, determined using diffusion-weighted magnetic resonance imaging, were also considered, along with relevant laboratory data.

### 2.3 Outcomes

The primary outcomes of this study was myocardial injury following AIS. The criteria for myocardial injury include the detection of an elevated cTn value above the 99th percentile upper reference limit (URL) (McCarthy et al., 2019). The injury is considered acute if there is a rise and/or fall of cTn values (>20%) (Thygesen et al., 2018). While stable elevation of cTnT levels may occur in various chronic cardiac conditions, a dynamic change in cTnT levels is specific to acute, evolving myocardial injury (Mahajan and Jarolim, 2011).

### 2.4 Statistical analysis

Statistical analysis was performed using SPSS version 27.0 and R Studio version 4.3.1. The Kolmogorov-Smirnov test and Levene's test were used to evaluate the normality and homogeneity of variance of continuous variables, respectively. Normally distributed data are presented as mean ± standard deviation (x ± s) and were compared using the independent samples *t*-test. Non-normally distributed data are expressed as median (M) with interquartile range (IQR) M(P25, P75) and compared using the Mann–Whitney *U*-test. Categorical variables were analyzed using the chi-square test. Variables showing significant differences between groups and potentially influencing myocardial injury were included in univariate logistic regression analysis. Variables with *P* < 0.05 in the univariate analysis were subjected to least absolute shrinkage and selection operator (LASSO) regression to identify non-zero coefficients, followed by multivariate logistic regression to determine independent risk factors for myocardial injury. These factors were further evaluated using receiver operating characteristic (ROC) curve analysis. Spearman's correlation was applied to assess the relationship between myocardial injury and the identified independent risk factors and to evaluate their diagnostic value.

## 3 Results

### 3.1 Baseline characteristics

A total of 490 patients with AIS complicated by DM were included in this study. Among these, 266 patients did not develop myocardial injury, while 194 patients exhibited myocardial injury. Patients in the myocardial injury group were older and had a higher BMI compared to those in the non-myocardial injury group. Furthermore, the myocardial injury group had a higher prevalence of comorbidities, more severe neurological deficits, and a greater incidence of insular cortex lesions. A higher proportion of patients in the myocardial injury group had a history of antiplatelet, anticoagulant, and lipid-lowering medication use. They also received reperfusion therapies, including thrombolysis and thrombectomy, more frequently than the non-myocardial injury group.

In terms of laboratory findings, patients with myocardial injury had significantly higher levels of cTnT, peak brain natriuretic peptide precursor (Peak NT-ProBNP), creatine kinase (CK), creatine kinase isoenzyme (CK-MB), neutrophil (N) count, C-reactive protein (CRP), uric acid (UA), cystatin C (CysC), aspartate aminotransferase (AST), and homocysteine (HCY). Conversely, they had lower levels of hemoglobin (Hb), lymphocyte (L) count, triglycerides, and albumin (ALB) compared to the non-myocardial injury group (Table 1).

**Table 1:** Baseline characteristics of the non-myocardial injury group and myocardial injury group.

**Variables**	**Non-myocardial injury group (*n* = 266)**	**Myocardial injury group (*n* = 194)**	***P*-value**
Age (yr)	65.00 (56.00, 72.00)	70.00 (62.00, 77.00)	<0.001
BMI (kg/m^2^)	25.39 (23.38, 27.45)	24.48 (22.58, 26.53)	0.014
**Gender** ***n*** **(%)**			0.721
F	92 (34.59)	64 (32.99)	
M	174 (65.41)	130 (67.01)	
**Conventional risk factors**, ***n*** **(%)**			
Hypertension	184 (69.17)	150 (77.32)	0.053
Smoking	139 (52.26)	106 (54.64)	0.613
Drinking	152 (57.14)	118 (60.82)	0.428
**Comorbidities** ***n*** **(%)**			
CHD	30 (11.28)	69 (35.57)	<0.001
Prior stroke	71(26.69)	73(37.63)	0.012
**Neurological status**			
NIHSS score	2.00 (1.00, 3.00)	4.00 (2.00, 8.00)	<0.001
Insular cortical lesions, *n* (%)	20 (7.52)	72 (37.11)	<0.001
**Previous medications**, ***n*** **(%)**			
Antiplatelet drugs	100 (37.59)	96 (49.48)	0.011
Anticoagulant drugs	9 (3.38)	17 (8.76)	0.014
β-Blocker	13 (4.89)	14 (7.22)	0.294
Statins	99 (37.22)	94 (48.45)	0.016
**Therapy methods** ***n*** **(%)**			<0.001
Traditional drugs	228 (85.71)	134 (69.07)	
Thrombolysis	30 (11.28)	48 (24.74)	
Thrombectomy	8 (3.01)	12 (6.19)	
**Main laboratory findings**			
Peak hs-TnT (ng/L)	8.82 (7.10, 10.60)	22.85 (17.55, 42.90)	<0.001
Peak NT-proBNP (pg/mL)	51.09 (34.67, 107.70)	395.60 (89.38, 1137.50)	<0.001
CK (U/L)	64.00 (47.00, 93.00)	77.00 (52.00, 129.00)	0.002
CKMB (ng/mL)	1.85 (1.39, 2.56)	2.15 (1.55, 2.78)	0.007
Hb (g/L)	138.00 (127.25, 150.00)	133.00 (115.19, 145.00)	<0.001
N (10^9^/L)	4.40 (3.52, 6.08)	5.29 (3.94, 8.14)	<0.001
L (10^9^/L)	1.60 (1.30, 2.10)	1.40 (1.00, 1.90)	<0.001
PLT (10^9^/L)	213.00 (179.00, 246.00)	206.50 (164.25, 249.25)	0.170
CRP (mg/L)	2.37 (1.00, 5.45)	11.55 (3.41, 34.48)	<0.001
FBG (mmol/L)	8.23 (6.47, 10.48)	8.66 (6.69, 11.33)	0.077
HbA1c (%)	7.80 (7.00, 9.20)	8.00 (6.90, 9.20)	0.723
TC (mmol/L)	4.46 (3.73, 5.34)	4.23 (3.40, 5.15)	0.072
TG (mmol/L)	1.56 (1.18, 2.31)	1.34 (1.04, 2.04)	0.005
LDL-C (mmol/L)	2.67 (2.04, 3.37)	2.47 (1.87, 3.25)	0.164
HDL-C (mmol/L)	0.92 (0.79, 1.12)	0.93 (0.82, 1.09)	0.860
UA (umo/L)	262.00 (216.50, 311.75)	274.00 (233.25, 354.00)	0.039
CysC (mg/L)	0.90 (0.78, 1.07)	1.04 (0.90, 1.39)	<0.001
ALB (g/L)	41.35 (38.73, 43.38)	38.65 (35.02, 41.90)	<0.001
AST (U/L)	16.00 (13.00, 20.00)	17.50 (14.00, 21.00)	0.009
HCY (umo/L)	14.29 (11.34, 18.42)	16.11 (12.69, 21.53)	0.006

BMI, body mass index; CHD, Coronary heart disease; NIHSS, National Institutes of Health Stroke Scale; Peak hs-TNT, Troponin; Peak NT-proBNP, Brain natriuretic peptide precursor; CK, creatine kinase; CK-MB, creatine kinase isoenzyme; Hb, hemoglobin; N, neutrophil; L, lymphocyte; PLT, platelet; CRP, C-reactive protein; FBG, fast blood glucose; HbA1c, Glycosylated Hemoglobin; TC, total cholesterol; TG, triglyceride; LDL-C, low-density lipoprotein cholesterol; HDL-C, high-density lipoprotein cholesterol; UA, uric acid; CysC, Cystatin C; ALB, albumin; AST, aspartate aminotransferase; HCY, homocysteine.

### 3.2 Logistic regression analyses and LASSO regression

**Figure 2:**
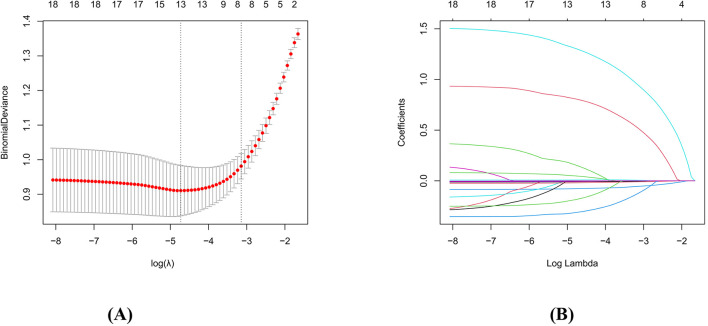
LASSO regression model screening risk factors of myocardial-injury. **(A)** LASSO regression model cross-validation plot. Draw a vertical line at the optimum with the minimum criterion and 1se of the minimum criterion. When λ =0.027020, we get 8 variables for further analysis. **(B)** Coefficient profile plot of risk factors. Finally, eight variables were selected at the optimal lambda, which is consistent with the results selected.

The indicators that showed significant differences between the two groups and were considered potential factors influencing myocardial injury were selected as independent variables, with myocardial injury as the dependent variable. Univariate logistic regression analysis identified age, BMI, CHD, stroke history, NIHSS score, insular cortical lesions, antiplatelet therapy, anticoagulant therapy, statin use, therapeutic methods, peak NT-proBNP, CK-MB, Hb, N count, CRP, TG, CysC, ALB, and HCY as factors significantly associated with myocardial injury (*p* < 0.05) (Table 2). These variables were then included in a LASSO regression analysis, which identified age, CHD, NIHSS score, insular cortex lesions, peak NT-proBNP, CRP, CysC, and ALB as risk factors for myocardial injury in patients with AIS and DM (Figure 2). And multivariate logistic regression analysis confirmed that CHD, NIHSS score, insular cortex lesions, peak NT-proBNP, and CRP were independent risk factors for myocardial injury in AIS patients with concurrent DM (Table 2).

**Table 2:** Logistic regression analysis.

**Variables**	**Univariate**		**Multivariate**	
	**OR (95%CI)**	**P**	**OR (95%CI)**	***P***
Age	1.04 (1.03 – 1.06)	<0.001	1.01 (0.98 – 1.03)	0.664
BMI	0.94 (0.89 – 0.99)	0.049	0.99 (0.91 – 1.07)	0.741
**Comorbidities**				
CHD	4.34 (2.69 – 7.02)	<0.001	2.52 (1.23 – 5.19)	0.012
Prior Stroke	1.66 (1.11 – 2.47)	0.013	1.46 (0.68 – 3.16)	0.334
**Neurological status**				
NIHSS score	1.19 (1.13 – 1.25)	<0.001	1.09 (1.03 – 1.15)	0.003
Insular cortical lesions	7.26 (4.23 – 12.47)	<0.001	4.56 (2.30 – 9.05)	<0.001
**Previous medications**				
Antiplatelet drugs	1.63 (1.12 – 2.37)	0.011	1.21 (0.42 – 3.50)	0.729
Anticoagulant drugs	2.74 (1.20 – 6.29)	0.017	0.77 (0.23 – 2.56)	0.676
Statins	1.59 (1.09 – 2.31)	0.016	0.71 (0.23 – 2.15)	0.541
**Therapeutic methods**				
Thrombolysis	2.72 (1.65 – 4.50)	<0.001	1.40 (0.68 – 2.86)	0.358
Thrombectomy	2.55 (1.02 – 6.40)	0.046	0.95 (0.24 – 3.78)	0.947
**Laboratory results**				
Peak NTproBNP (pg/mL)	1.01 (1.01 – 1.01)	<0.001	1.01 (1.01 – 1.01)	<0.001
CK (U/L)	1.00(1.00 – 1.00)	0.360		
CKMB (ng/mL)	1.23 (1.05 – 1.45)	0.012	1.18 (0.96 – 1.46)	0.117
Hb (g/L)	0.99 (0.98 – 0.99)	0.002	1.00 (0.99 – 1.02)	0.658
N (10^9^/L)	1.04 (1.01 – 1.07)	0.025	1.01 (0.98 – 1.04)	0.448
L (10^9^/L)	1.03 (0.95 −1.12)	0.483		
CRP (mg/L)	1.05 (1.03 – 1.06)	<0.001	1.02 (1.01 – 1.04)	0.005
TG (mmol/L)	0.82 (0.68 – 0.98)	0.027	0.92 (0.74 – 1.15)	0.468
UA (umo/L)	1.00 (1.00 – 1.00)	0.084		
CysC (mg/L)	3.01 (1.87 – 4.84)	<0.001	1.46 (0.75–2.82)	0.263
ALB (g/L)	0.91 (0.87 – 0.94)	<0.001	0.99 (0.94 – 1.05)	0.790
AST (U/L)	1.01 (1.00 – 1.02)	0.097		
HCY (umo/L)	1.03 (1.01 – 1.05)	0.021	1.01 (0.97 – 1.05)	0.597

### 3.3 Correlation analysis

cTnT was significantly positively correlated with the following risk factors (Coronary heart disease: ρ = 0.302, *p* < 0.01; Insular cortical lesions: ρ = 0.329, *p* < 0.01; Peak NT-proBNP: ρ = 0.538, *p* < 0.01; C-reactive protein: ρ = 0.436, *p* < 0.01; NIHSS score: ρ = 0.378, *p* < 0.01). In this study, cTnT was most strongly correlated with Peak NT-proBNP (Table 3).

**Table 3:** Correlation between relevant risk factors and cardiac troponin T.

**Variables**	**ρ**	***P***
Coronary heart disease	0.302	0.01
Insular cortical lesions	0.329	0.01
Peak NT-proBNP	0.538	0.01
C-reactive protein	0.436	0.01
NIHSS score	0.378	0.01

### 3.4 ROC curve analysis

**Figure 3:**
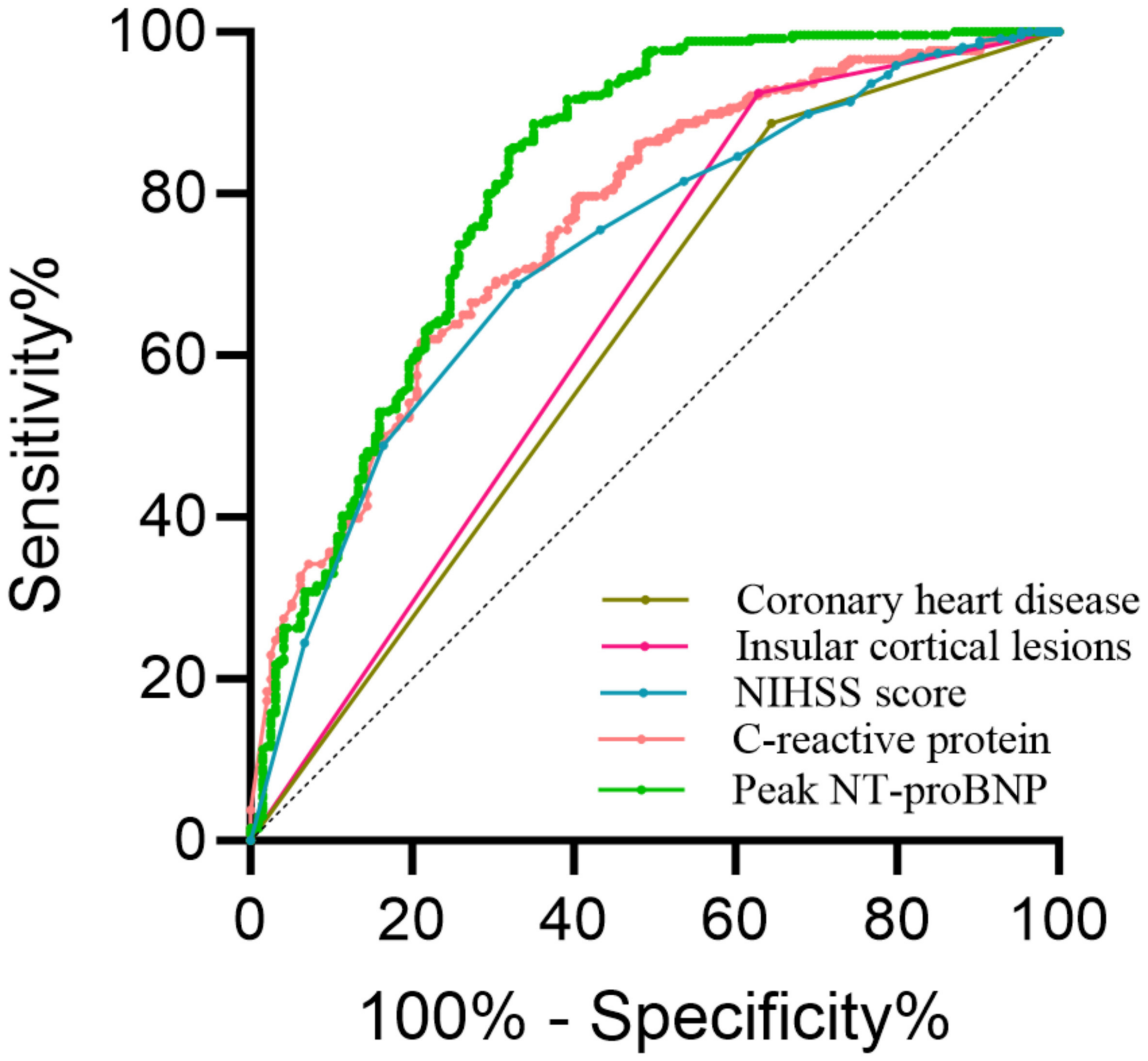
ROC curve analyzing the following indicators.

Patients with AIS and DM exhibit an elevated risk of myocardial injury. CHD, Insular cortex lesions, NHISS Score, peak NT-proBNP, and CRP were identified as independent risk factors for myocardial injury in this cohort. To assess the diagnostic utility of these factors, receiver operating characteristic (ROC) curve analysis was performed to determine the area under the curve (AUC) for each variable. The AUC values were as follows: 0.621 for CHD, 0.648 for insular cortex lesions, 0.726 for NIHSS score and a cut-off value of 2.5, 0.816 for peak NT-proBNP and a cut-off value of 193.5, and 0.764 for CRP and a cut-off value of 3.125 (Figure 3).

### 3.5 Subgroup analysis of reperfusion therapy

**Figure 4:**
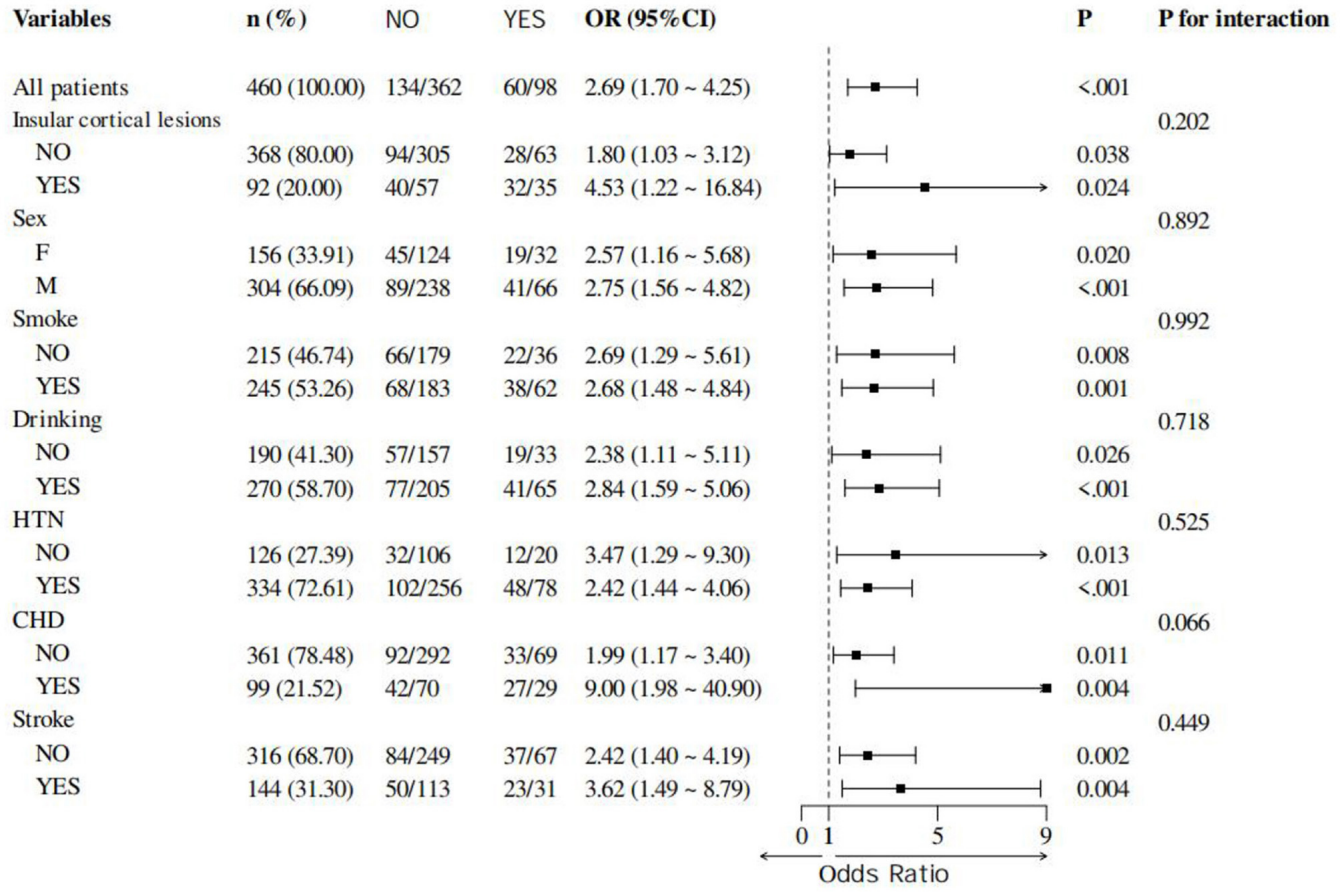
Forest plot of odds ratios (OR) for myocardial injury in different subgroups.

Subgroup analyses revealed a statistically significant overall effect of reperfusion therapy on myocardial injury across the entire study population. And the effect of reperfusion therapy on myocardial injury was consistent across all seven pre-specified subgroups, with no interaction (all interaction *p*-values >0.05). However, it did not affect the relationship between the two (Figure 4).

## 4 Discussion

Previous studies have reported a myocardial injury incidence of 27.2% in IS patients. In contrast, this study found a higher incidence of 39.59%, which may be attributed to the inclusion of DM patients. DM is an independent risk factor for cardiovascular and cerebrovascular diseases and may increase the risk of post-stroke myocardial injury through mechanisms such as cardiac metabolic dysfunction, oxidative stress, and inflammation. This could explain the higher myocardial injury incidence observed in this study.

In this single-center retrospective cohort study, CHD, insular cortex lesions, peak NT-proBNP, CRP, and NIHSS score were identified as significant risk factors for myocardial injury, with these factors positively correlated with the severity of myocardial injury.

A previous study by Song et al. (2024) investigated the relationship between CHD and post-stroke myocardial injury, identifying a history of CHD as one of the significant causes of myocardial injury after stroke. The underlying mechanism may involve the presence of coronary atherosclerosis or stenosis in CHD patients, leading to inadequate myocardial oxygen supply, which further increases the risk of myocardial injury under the stress condition of acute ischemic stroke (Song et al., 2024). CHD is closely associated with systemic inflammation, which plays a key role in atherosclerosis and thrombosis (Wang et al., 2024). After acute ischemic stroke, inflammation may exacerbate the existing myocardial injury (Wang et al., 2024). Additionally, CHD patients often experience reduced myocardial energy metabolism efficiency, and diabetes can worsen endothelial dysfunction and coronary microvascular impairment, further increasing the risk of post-stroke myocardial injury (Wang et al., 2024; Sourour et al., 2024). In this study, CHD was found to be a risk factor for myocardial injury, with CHD patients being more likely to develop myocardial injury (OR 2.52, 95% CI 1.23–5.19), consistent with the findings of Song et al. (2024). Krause et al. (2017) conducted foundational research demonstrating that the insular cortex is a critical regulatory center of the central autonomic network, involved in the modulation of both sympathetic and parasympathetic control of the heart in response to emotional and physical stress. Scheitz et al. (2021) utilized voxel-based lesion-symptom mapping and confirmed that right dorsolateral anterior insular cortex lesions are associated with changes in post-stroke cTnT levels. In this study, patients with insular cortex lesions exhibited a higher incidence of myocardial injury. Moreover, in the subgroup analysis of reperfusion therapy, patients with insular lesions had a higher rate of reperfusion treatment, suggesting that their condition may be more severe.

The severity of stroke and post-stroke mortality have been linked to various biomarkers, with different natriuretic peptide levels correlating with stroke severity and mortality (Johansen et al., 2024). In a study involving 788 ischemic stroke patients, brain natriuretic peptide (BNP) levels were significantly associated with 90-day post-stroke mortality, while another study found BNP to be independently correlated with functional outcomes at 90 days (Hellwig et al., 2021; Hatab et al., 2023; Nigro et al., 2014). Research by Nigro et al. (2014) revealed that BNP levels were associated with recurrent vascular events after stroke, and even after adjusting for heart failure and cardioembolic stroke etiology, NT-proBNP remained an independent predictor of post-stroke mortality. In this study, peak NT-proBNP was identified as a risk factor for myocardial injury following stroke, with a strong correlation, consistent with previous findings.

Recent experimental studies support the critical role of inflammatory responses in the pathophysiology of SHS (Fan et al., 2024). In stroke mouse models, cardiac dysfunction is primarily accompanied by systemic inflammation, upregulation of pro-inflammatory cytokines in myocardial tissue, and infiltration of macrophages into the heart (Yan et al., 2020; Vornholz et al., 2021). Post-stroke adverse outcomes are linked to blood-brain barrier disruption, which may promote neutrophil and macrophage infiltration into ischemic brain tissue, a process that plays a crucial role in maintaining the integrity of the neurovascular unit in ischemic stroke models (Barca et al., 2021). Furthermore, excessive sympathetic nervous system activation can trigger the migration of inflammatory cells from the bone marrow or spleen. Previous studies have indicated that the spleen plays a key role in the subacute and long-term outcomes of stroke-induced heart syndrome, ultimately contributing to chronic remodeling (Scheitz et al., 2022). In this study, we observed that inflammation is correlated with myocardial injury.

The NIHSS score is a widely used tool for assessing neurological function in acute stroke patients and correlates with stroke severity. A prospective study has demonstrated that NIHSS scores are associated with elevated cTnT levels (OR, 1.05; 95% CI, 1.02–1.07) (Ahn et al., 2023). In this study, patients with myocardial injury had higher NIHSS scores, reflecting more severe neurological deficits.

The risk factors identified in this study are well-supported by existing evidence; however, several limitations should be acknowledged. (1) This study is a single-center, retrospective analysis with a relatively small sample size, which may limit the generalizability of the findings. Future multicenter, prospective studies are needed to further explore the potential risk factors involved. (2) The study lacks quantitative imaging data, which precludes an examination of the impact of stroke lesion volume on myocardial injury. Future research will aim to incorporate such data to provide a more comprehensive analysis.

## 5 Conclusion

The results of this study indicate that CHD, insular cortex lesions, peak NT-proBNP, CRP, and NIHSS score are independent risk factors for myocardial injury in patients with AIS and DM. These findings provide valuable evidence for the early identification of risk factors for myocardial injury and for the implementation of early interventions.

## Data Availability

The raw data supporting the conclusions of this article will be made available by the authors, without undue reservation.
